# Non-Medical Activities in Dementia Care in Germany: Use and Experienced Effects

**DOI:** 10.1177/21501319251390081

**Published:** 2025-11-04

**Authors:** Francisca S. Rodriguez, Nadja Ziegert, Sabrina D. Ross

**Affiliations:** 1 German Center for Neurodegenerative Diseases (DZNE), Greifswald, Germany

**Keywords:** dementia, Alzheimer’s, dementia care, health service utilization, non-pharmacological, psychosocial, intervention

## Abstract

Previous studies have shown benefits through non-pharmacological interventions for people with dementia. Non-medical activities (i.e., activities outside the medical sector) may have similar effect. As little is known about the use and perceived effects of non-medical activities in dementia care, this study’s aim was to obtain population-based descriptive information. A survey in the form of structured interviews was conducted with n = 134 stakeholders. Participants used on average 17.7 non-medical activities (i.e., social, leisure, and household activities). They reported perceiving effects for, on average, 85.1% of the activities, which were mostly effects on improvements in wellbeing, activation, and social health. Overall, a higher use of non-medical activities was significantly associated with perceiving more effects, especially on cognition and preserving abilities, and perceiving not knowing an activity as a barrier. However, this differed by stakeholder group: Perceiving effects on cognition was only significant for caregiving professionals. Further, for this group, feeling sufficiently trained for dealing with dementia and self-organizing/ self-financing activities was associated with a higher use. Overall, the results indicate that non-medical activities are an important component of dementia care that seem to come with important benefits.

## What the Paper Adds to Existing Literature

- As participants use many non-medical activities and perceive effects for most of them, these activities may bring benefits for dementia patients that should be provided in a sustainable way.
- The observation that people with dementia and family caregivers reported engaging in fewer activities than professional caregivers indicates the existence of unknown barriers for this group.

## Applications of Study Findings to Gerontological Practice, Policy, and/or Research

- The findings propose that non-medical activities may bring additional effects for well-being and maybe even the course of dementia symptoms, which will have to be explored in future research.
- Utilization of non-medical activities could be simple strategy for symptom management that is worth exploring in more detail in trials.

## Background

With more than 57 million people worldwide currently living with dementia and numbers raising to over 150 million within the next 30 years,^1^ dementia is one of the greatest global challenges. As there is still no cure against dementia that predominantly affects older adults, it is the goal to optimize dementia care by slowing the course of the disease, reducing the symptoms, and improving wellbeing. Pharmacological solutions can provide some relief, but they have not consistently shown to alter the course of the disease and are definitely not sufficient to address the broad range of challenges that people with dementia and their caregivers face. Hence, non-pharmacological interventions are increasingly recognized for their potentials.

Among non-pharmacological interventions, cognitive stimulation,^2^ physical activity,^3^ music therapy,^4^ and snoezeln (i.e., multi-sensory stimulation of the senses)^5^ have been investigated in many studies and evidence shows important benefits for people with dementia. In fact, a sufficient amount of evidence has been produced so that national guidelines for treating dementia recommend non-pharmacological interventions. For example, the *German S3 Guidelines Dementia* that explicitly recommends the use of cognitive stimulation, physical activity, music therapy, aromatherapy, and snoezeln as well as further interventions upon particular indication.^6^ While some types of interventions, such as cognitive stimulation for improving cognitive abilities, exercise for maintaining daily functioning, and music therapy for reducing behavioral issues^7^ are already widely recognized, other types of intervention have just started to receive attention. For instance, studies demonstrated that animal assisted therapy reduced aggressive behavior and anxiety^8^; playing games, singing, and massages relieved pain,^9^ outside activities (e.g., water sports, excursions) improved psychological wellbeing,^10^ and yoga-related mind-body therapies improved cognitive abilities.^11^ Hence, there are many promising potentials. Nonetheless, and sometimes despite of recommendations, non-pharmacological interventions are not yet part of standardized treatment.^12^ Yet, in some countries, availability and uptake is increasing and prescribing such interventions is starting to be explored. One aspect is certainly that increasing the use of non-pharmacological interventions would enhance the burden of an already highly strained health care sector. Implementing non-pharmacological interventions as a form of home-based activities, for instance as leisure activities, would be an alternative approach. As these are carried out outside the medical sector (i.e., without a therapist or similar), we will refer to them as “non-medical” within this paper. Such non-medical strategies could avoid additional burden in the medical sector.

Non-medical activities in dementia care can have effects way beyond sustaining and supporting people with dementia in their daily chores. They can address a variety of cognitive, emotional, and psychological aspects,^13^ so that home-based activities can possibly be a resource for wellbeing.^14^ However, there is hardly any information available on how these type of activities are being used in dementia care. As they are not part of standard care, there exists no medical records on their use. Nonetheless, it is important to evaluate the use of such activities in dementia care in order to obtain a better understanding of their relevance for symptom management.

The aim of the study was to get a first impression of the use of non-medical activities, including household activities, leisure activities, and social activities, in dementia care and the perceived effects of them. To obtain a comprehensive overview of the current state of use, we obtained data from (i) people with dementia and family caregivers, (ii) caregiving professionals, and (iii) people otherwise involved in dementia care (e.g., counselors, therapists, physicians) in structured interviews. Moreover, we analyzed in an explorative analysis what aspects (age, sex, education, feeling sufficiently trained, perceived barriers, activity provider, financing, perceived effects) predict the use of non-medical activities.

## Methods

### Study Design

This study was a cross-sectional survey in the form of structured interviews with different stakeholders with the purpose of obtaining population-based descriptive information.

### Sample

Participants were recruited in 2021 in Germany via strategic sampling. As a first step, we contacted physicians, therapists, and nursing homes in our state and subsequently all other states of Germany. In a second step, we systematically contacted nursing care networks, dementia networks, support groups, geriatric care working groups, professional associations, and organizers of nursing training courses in every state in Germany. Information on the study was distributed via email, flyers, and on the phone. Inclusion criteria were an age of at least 18 years, sufficient German, hearing, vision, and mental abilities as well as the capacity to consent (e.g., not in delirium or clouded consciousness). Excluded were people that had hearing, vision, language, or mental capacities that were so severe that conducting the interviews would have been difficult. Those interested in participating were informed about the aims and the process of the study, and had opportunity to ask questions. If they signed an informed consent, an appointment for a telephone-based or face-to-face interview was made. If relevant, informed consent from the Legally Authorized Representative (LAR) was obtained as well. The interviews were structured (see following section) and were conducted by one of the authors (FR, SR, NZ). Of the n = 134 people who participated, n = 50 were people with dementia (PwD) and family caregivers (n = 10 people with dementia, n = 40 were family caregivers), n = 42 were “caregiving professionals” (n = 20 caregiving professionals, n = 22 personal assistants), and n = 42 were “otherwise involved” in dementia care (n = 26 therapists, n = 13 counselors, n = 3 physicians). As 1 person with dementia and 1 caregiving professional provided insufficient information, the sample available for analyzing non-medical activities was n = 132.

### Use of Non-Medical Activities

In a structured interview, participants answered questions on experience of dementia care and reported on their use of activities. Each participant reported on their own use and practice. While PwD and family caregivers reported on their use, professional caregivers and those otherwise involved reported on the use of activities in their work with all their patients. A list of 51 activities was derived by screening (i) the current guidelines, (ii) scientific literature, (iii) local dementia care programs, and (iv) communication in previous studies. The final list comprised 15 therapies, 4 education programs, and 32 non-medical activities. In this analysis, we investigate only the non-medical activities, which were comprised of 5 household activities (cooking, baking, laundry, cleaning, fixing broken things), 8 social activities (making phone calls, visits, excursions, volunteering, using visitation services, using phone services, receiving neighborhood assistance, attending club meetings), and 19 leisure activities (gardening, playing card/board games, reading, watching TV, puzzling (e.g., crossword puzzles, Sudoku), going to cinema/ museum/ theater, needlework (e.g., crocheting, knitting, or embroidery), drawing, crafts, singing, playing a musical instrument, dancing, listening to music, keeping pets, physical activity, attending religious events, spending time in nature, learning/using a foreign language, traveling/vacation. Household activities were included as there is a tendency by caregivers and nursing homes to take over these activities from PwD. However, household activities can be a useful strategy for promoting wellbeing, activation, social integration, and experiencing appreciation, even when the person is not able to do so independently anymore. Hence, actively carrying out household activities may be beneficial for PwD. Use of each of the 32 activity was assessed by the question whether the participants employed the activities, yes (1) or no (0). For analysis, we added up the answers (score range 0-32). Even though we should not necessarily expect a correlation of use of activities, the internal consistency is relatively high (total score: Cronbach’s alpha (α) = 0.865; specifically for household activities α = 0.763, social activities α = 0.652, and leisure activities α = 0.831).

### Predictors and Confounders

Age, sex, and education were reported by the participant. For purpose of analysis, education was categorized as “high” for having completed university or college and “low” for less than that. Feeling trained was assessed with the question, “Do you feel you are sufficiently trained to deal [with people] with dementia?” (yes (1)/no (0)).

#### Providers

Providers of activities were assessed with the question, “Who offered the activity?” Answers were categorized into (i) self-organized, (ii) organized by an external caregiver, or (iii) organized by an external party (e.g., association, medical facility) for each activity. We counted the number of activities in each category for each participant. To reflect the average percentage of activities that were organized by oneself/ external caregiver/ external party, for each participant, the 3 scores were divided by the total number of activities the participant used and multiplied by 100.

#### Financing

Type of financing was assessed with the question, “Who paid for the activity?” Answers were categorized into (i) for free, (ii) financed privately, (iii) services or benefits by health and/or nursing care insurances, and (iv) financed through providers (e.g., nursing home, day care, associations) for each activity. Sum scores for each category were created for each participant. Again, these scores were divided by the total number of activities and multiplied by 100 to reflect average percentages.

#### Perceived Effects

Perceived effects of the activities were documented as reported by the participants to the question, “What positive effects do you perceive for this activity?” First, we assigned a score on whether an effect was perceived (yes (1)/ no (0)). Again, we added up these scores over all activities and transformed it to a percentage score. Second, to specify the types of effect that were perceived, 2 independent blinded raters (FR, SR) attributed categories to the reported effects. The average incongruence between the category ratings of the 2 raters was 14.5%. Incongruences were discussed in team meetings (FR, SR, and NZ) until a consensus was achieved. The final categories were: improved cognition, activating, preserving abilities, wellbeing, appreciation, relaxation, and improved social health. For each type of effect, we added the number of activities for which this effect was reported. These scores were also divided by the total number of activities of each participant and multiplied by 100 to reflect average percentage of activities for which the participants perceived this effect.

#### Barriers

Barriers were assessed with the question, “What prevents you from using activities?” The multiple answer categories (none/ accessibility/no time/no money/organizational effort/ personal attitude/activity unknown/ content unknown/benefits unknown/feeling insecure among strangers/stigma) were each coded with yes (1) or no (0), respectively.

### Analysis

Data analysis was conducted using Stata 16.0 and a significance level of 0.05. For continuous predictor variables, we added 1 and log transformed them to come as close to a normal distribution as possible. To estimate univariate associations between use of non-medical activities and possible predictors, we used Kruskal-Wallis test (categorical variables) or Spearman’s rank correlation (continuous variables); both statistical tests tolerate distributions that are not exactly normal distributions.

## Results

Participants were on average 55.2 years old (SD = 14.3, range 26-85 years). Seventy-five percent were female, 58.9% highly educated, and the majority (82%) felt sufficiently trained for dementia.

On average, 17.7 (SD = 6.3, range 3-32) non-medical activities were reported to be used. Of those, 11.1 (SD = 4.3) were leisure activities, 2.7 (SD = 1.7) were household activities, and 3.9 (SD = 1.7) were social activities. “PwD & family caregivers” used on average 3.1 activities less than caregiving professionals and 7.6 activities less than “otherwise involved” stakeholders (see Supplementary File, Table S1). This corresponds to about 2 household, 2 social, and 4 leisure activities. For most activities, fewer “PwD & family caregivers” than participants from the other groups reported using them. No difference was observed for “listening to music,” “reading,” “spending time in nature,” “cleaning,” and “puzzling,” while “watching TV” and “traveling” was used by more “PwD & family caregivers” than participants from the other groups (see Supplementary File, Table S1).

**Figure 1.**
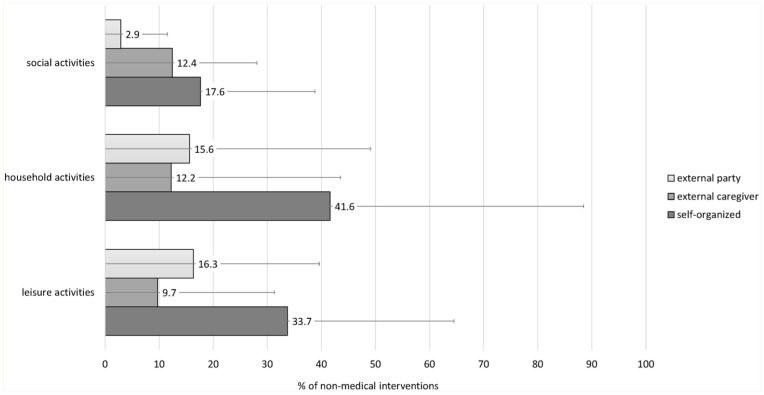
Financing of non-medical activities (social activities, household activities, leisure activities) used by the participants. Average percentages with standard deviations as error bars.

On average, 32.8% (SD = 26.5%) of the activities were organized privately, while 9.3% (SD = 19.5%) were organized by an external caregiver, and 16.1% (SD = 19.3%) were provided by an external party such as an association. A much smaller percentage of social activities (17.6%) than household (41.6%) or leisure activities (33.7%) were privately organized, or provided for by external parties (2.9% of social, 15.6% of household, and 16.3% of leisure activities). Regarding the costs, participants reported that 39.4% (SD = 37.9%) of the non-medical activities were financed privately, 13.7% (SD = 27.3%) were financed through the provider (other than self), 12.1% (SD = 23.1%) were services or benefits by insurances, and 2.5% (SD = 8.4%) were for free. Social activities, compared to leisure or household activities, received less financing by insurances or providers (see Figure 1).

Engaging in activities is hindered by barriers, as 82.8% of our participants reported. A total of 32.8% saw a lack of money as a barrier, 30.6% the organizational effort, 28.4% accessibility, 20.9% not knowing the benefits, 17.2% a lack of time, 13.4% the personal attitude, 12.7% not knowing about the activities, 8.2% not knowing the content of activities, 7.5% stigma towards dementia, and 5.9% feeling insecure among strangers.

### Perceived Effects

**Figure 2.**
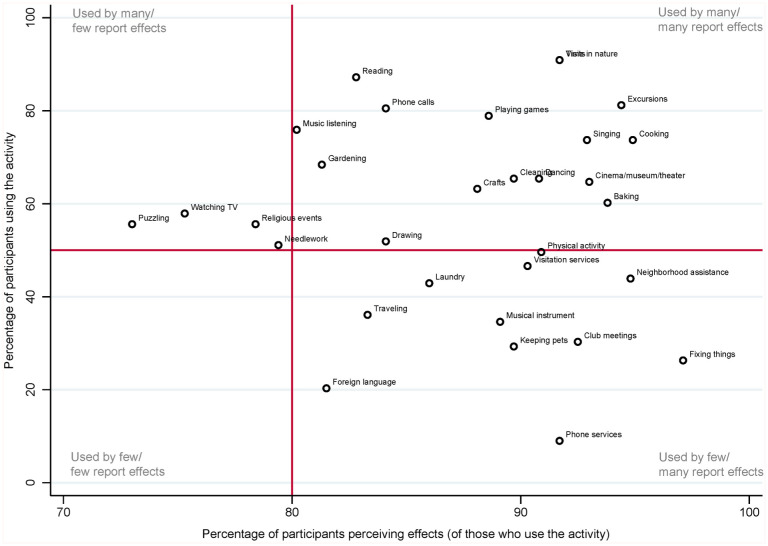
Percentage of participants using a non-medical activity and how many perceived an effect for it.

Participants reported that they perceived effects for, on average, 85.1% (SD = 28.1%) of the activities. This number was slightly lower for household activities (M = 74.5%, SD = 42.8%) than for social (M = 81.9%, SD = 33.8%) and leisure activities (M = 84.7%, SD = 32.7%). Activities for which most participants reported effects were cooking, neighborhood assistance, and fixing broken things (see Figure 2 and Supplementary File, Table S2). Activities for which the fewest participants reported effects were watching TV, puzzling, and needlework. PwD and family caregivers reported less frequently perceiving effects than caregiving professionals or “otherwise involved” participants, in particular on preserving abilities, activation, and improved social health (see Supplementary File, Table S3).

**Figure 3.**
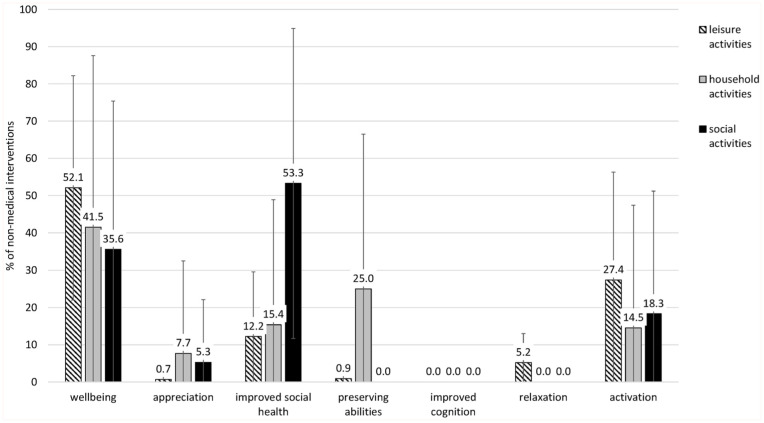
Perceived effects of non-medical activities (social activities, household activities, leisure activities) used by the participants. Average percentages with standard deviations as error bars.

Concerning the types of effect, participants perceived improvement in wellbeing through, on average, 49.6% (SD = 26.9%) of the non-medical activities, activation through 24.0% (SD = 24.9%), improved social health through 23.7% (SD = 18.9%), preserving abilities through 5.3% (SD = 8.1%), relaxation through 3.4% (SD = 5.0%), appreciation through 3.2% (SD = 7.4%), and improved cognition through 0.9% (SD = 2.0%) of the non-medical activities. Figure 3 shows the perceived effects separately for leisure, household, and social activities. Improved wellbeing was perceived for most activities. Besides wellbeing, for leisure activities, the most frequently reported effect was activating (M = 27.4%, SD = 28.9%), for household activities, preserving abilities (M =25.0%, SD = 41.5%), and for social activities, improved social health (M = 53.3%, SD = 41.6%).

### Predictors of Use

In univariate analyses, people with dementia and family caregivers reported using significantly less non-medical activities than caregiving professionals and those “otherwise involved” in dementia care. Using more non-medical activities was significantly associated with perceiving not knowing an activity as a barrier (see Table 1), and perceiving more effects, especially on cognition and preserving abilities (see Table 2). Analyzing only “PwD & family caregivers,” perceiving more effects on cognition became non-significant (see Table 2). Interestingly older, compared to younger, “PwD & family caregivers” used significantly more activities (see Table 2; age <60. M = 12.7 activities, age 60-70: M = 14.5 activities, age >70: M = 15.6 activities).

**Table 1.** Univariate Associations Between the Number of Non-medical Activities Used With Participant Characteristics and Perceptions (Categorical Variables).

Participant characteristics/ perceptions	All participants (n = 132)		People with dementia and family caregivers (n = 49)		Caregiving professionals (n = 41)		“Otherwise involved” stakeholders (n = 42)	
	M (SD)	x² (*P*)	M (SD)	x² (*P*)	M (SD)	x² (*P*)	M (SD)	x² (*P*)
Group								
PwD and family caregivers	14.3 (4.6)	**30.945 (<.001)**						
Caregiving professionals	17.4 (4.7)							
“Otherwise involved”	21.9 (6.9)							
Sex								
Female	17.7 (6.4)	0.103 (.748)	13.9 (5.0)	1.224 (.269)	17.8 (4.7)	0.563 (.453)	21.2 (6.8)	2.734 (.098)
Male	17.7 (6.0)		15.2 (3.3)		16.5 (4.8)		26.0 (6.6)	
Education								
Low	16.4 (4.9)	3.792 (.052)	13.9 (4.5)	0.849 (.357)	17.1 (3.6)	0.302 (.582)	19.5 (4.9)	**4.847 (.038)**
High	18.5 (6.9)		14.7 (4.7)		17.6 (5.2)		23.3 (7.7)	
Feel sufficiently trained for dementia								
No	17.4 (7.3)	1.467 (.226)	13.8 (3.4)	0.420 (.517)	11.4 (4.6)	**8.497 (.004)**	23.5 (5.7)	0.390 (.532)
Yes	19.1 (6.1)		14.6 (4.9)		18.7 (3.8)		21.5 (7.2)	
Perceived barriers								
None								
No	18.2 (6.4)	2.452 (.117)	14.7 (4.4)	1.002 (.317)	17.8 (4.6)	0.133 (.716)	22.1 (7.0)	1.259 (.262)
Yes	15.4 (5.3)		12.6 (5.5)		16.8 (4.9)		17.5 (3.5)	
Accessibility								
No	17.8 (6.1)	0.073 (.786)	14.8 (4.5)	0.576 (.448)	17.5 (4.9)	0.036 (.849)	22.0 (7.1)	0.056 (.813)
Yes	17.4 (6.7)		13.3 (4.8)		17.3 (4.1)		21.6 (6.9)	
No time								
No	17.4 (6.4)	0.931 (.335)	14.2 (4.8)	0.093 (.760)	17.6 (4.8)	0.456 (.499)	21.4 (7.4)	0.635 (.426)
Yes	19.0 (5.6)		15.0 (2.6)		16.4 (4.4)		23.7 (4.7)	
No money								
No	17.7 (6.4)	0.002 (.961)	14.5 (4.7)	0.237 (.626)	17.5 (5.2)	0.100 (.752)	22.1 (7.0)	0.151 (.698)
Yes	17.7 (6.1)		13.9 (4.4)		17.3 (3.6)		21.4 (7.0)	
Organizational effort								
No	17.4 (6.3)	0.136 (.712)	14.3 (4.9)	0.004 (.949)	17.4 (5.1)	0.005 (.941)	20.8 (7.0)	2.431 (.119)
Yes	18.3 (6.3)		14.4 (3.9)		17.5 (3.7)		24.5 (6.2)	
Personal reasons								
No	17.5 (6.2)	0.813 (.367)	14.3 (4.7)	0.015 (.903)	16.9 (4.6)	1.350 (.245)	21.3 (7.1)	2.122 (.145)
Yes	19.1 (6.4)		14.4 (4.3)		20.2 (4.9)		27.0 (1.4)	
Activity unknown								
No	17.0 (6.2)	**11.607 (.001)**	13.9 (4.5)	**5.044 (.025)**	17.2 (4.8)	1.207 (.272)	20.9 (7.2)	3.333 (.068)
Yes	22.1 (4.8)		18.4 (3.4)		19.5 (3.0)		25.8 (3.8)	
Contents unknown								
No	17.5 (6.0)	1.703 (.192)	14.1 (4.5)	1.496 (.221)	17.4 (4.8)	0.001 (.980)	21.6 (7.2)	0.470 (.493)
Yes	19.8 (6.9)		17.3 (5.4)		17.3 (4.2)		24.3 (4.2)	
Benefits unknown								
No	17.2 (6.3)	2.284 (.131)	14.4 (4.8)	0.001 (.979)	17.5 (4.7)	0.002 (.965)	20.9 (7.2)	1.588 (.208)
Yes	19.6 (5.5)		14.2 (3.6)		17.0 (5.8)		23.6 (6.3)	
Feel insecure among strangers								
No	17.8 (6.2)	1.561 (.212)	14.3 (4.7)	0.064 (.801)	17.7 (4.7)	1.066 (.302)	22.2 (6.5)	0.636 (.435)
Yes	15.3 (6.5)		15.0 (.0)		15.3 (4.9)		15.5 (14.8)	
Stigma								
No	17.8 (6.3)	0.411 (.522)	14.2 (4.7)	0.696 (.404)	17.5 (4.7)	0.174 (.676)	22.2 (6.5)	0.636 (.425)
Yes	16.2 (6.2)		16.3 (1.5)		16.5 (5.3)		15.5 (14.8)	

Abbreviations: Bold, statistically significant with *p*<0.05; M, mean; *P*, level of significance; SD, standard deviation; x², chi square.

**Table 2.** Univariate Associations Between the Number of Non-medical Activities Used With Participant Characteristics, Participation Characteristics, and Effects Perceived (Continuous Variables).

Participant characteristics/ perceptions/ perceived effects	All participants (n = 132)		People with dementia and family caregivers (n = 49)		Caregiving professionals (n = 41)		“Otherwise involved” stakeholders (n = 42)	
	rho	*P*	rho	*P*	rho	*P*	rho	*P*
Age	−.082	.353	.317	.027	.241	.129	.048	.762
Provider: percentage of activities that were^a^								
Self-organized	−.044	.620	.068	.642	.357	**.022**	−.285	.067
External caregiver	−.071	.420	.108	.459	−.209	.191	−.145	.359
External party	−.044	.615	.148	.312	−.195	.221	−.125	.432
Financing: percentage of activities that were^a^								
For free	−.059	.497	−.100	.494	.159	.321	.120	.448
Privately financed	.057	.516	.118	.419	.319	**.042**	.252	.108
Services/benefits by insurances	−.028	.749	−.098	.500	.049	.757	−.598	**<.001**
Financed through providers	−.093	.288	.036	.808	−.144	.368	−.201	.203
Effect perceived, percentage of activities^a^	.221	**.011**	.144	.323	.064	.693	−.018	.908
Type of effect, percentage of activities^a^								
Wellbeing	.097	.269	.186	.201	−.208	.192	.015	.926
Appreciation	.053	.546	−.205	.157	.246	.122	−.094	.552
Improved social health	.149	.087	.074	.616	.209	.190	−.109	.489
Preserving abilities	.268	**.002**	.316	**.027**	.083	.607	.018	.912
Improved cognition	.234	**.007**	.061	.679	.369	**.018**	−.089	.572
Relaxation	.061	.486	.233	.107	−.045	.781	−.045	.777
Activation	−.146	.094	−.159	.273	−.266	.093	−.231	.142

a Percentage of all activities that the participant engaged in and (log + 1) transformed. B﻿old, statistically significant with *p*<0.05; *p*, level of significance; rho, spearman’s rank correlation coefficient.

Analyzing only caregiving professionals revealed a slightly different pattern: Using more non-medical activities was significantly associated with perceiving more effects on cognition, feeling sufficiently trained for dealing with dementia, a higher percentage of activities being self-organized or financed privately (see Tables 1 and 2). For example, caregiving professionals who reported self-organizing more than a third of the activities used on average M = 20.7 activities, while those who reported self-organizing less than one third used on average M = 16.5 activities. For privately financing activities, the averages were M = 21.4 versus M = 16.5.

For those “otherwise involved” in dementia care, the number of non-medical activities was only significantly associated with education and the percentage of activities financed through insurances (see Tables 1 and 2). Those who reported that more than one third of the activities were financially covered by insurances used significantly less non-medical activities (M = 16.5) than those who reported that less than one third were covered (M = 24.2).

## Discussion

The aim of the study was to investigate the use of non-medical activities in dementia care and the perceived effects through them. Results indicate that, on average, about 18 activities were being used, of which around 11 were leisure activities, 3 were household activities, and 4 were social activities. A great number of these activities were organized and financed privately, especially social activities. Nonetheless, with about fifteen percent of the activities of our participants, external providers such as associations and medical facilities provided and financed an important quantity of activities. The availability of such programs in the community for people with dementia has increased in the recent years,^15^ explaining why so many of our participants profited from them. Initiatives such as caregiver training programs and community centers could be useful to promote non-medical activities. Indeed, initiatives for dementia-friendly communities have started to bring together financial, social, and human capital to engage in the process of planning and implementing activity programs for and with people with dementia.^16^ Accordingly, it is to be expected that the share of activities provided by associations, communities, or similar facilities will increase in the coming years. For people with dementia still living at home, meeting centers (public or private) can be facilitators for participating in activities. However, such centers are not yet as available as needed; this might be particularly true for rural areas and laggard regions with weak infrastructure. The fact that most of the activities are organized and financed privately, as our results suggest, can lead to decreased participation in meaningful activities when someone either does not have much money or moves into a nursing home, where the possibilities (e.g., staff readiness, regulations) are restricted.^17^ Caregiving professionals could compensate for that, but also for them private initiative seems to be essential. Our analyses indicate that caregiving professionals who implemented more non-medical activities with their patients organized and financed a great part of them privately.

According to our participants, the most frequently reported barriers of engaging in non-medical activities were lack of money, the organizational effort, and accessibility. However, none of these significantly predicted the use of activities. On the contrary, those who perceived not knowing activities as barrier reported a significantly higher use. A possible explanation for that could be that individuals with a high motivation to engage in activities (i.e., higher overall use) also like to be informed about the types of activities available to them, while those with less motivation (i.e., lower overall use) only use of whatever happens to be at hand. Barriers that we did not ask about could play a role. Previous research shows that in particular enabling factors explain service use of people with dementia,^18^ which might be specific training of caregivers, local conditions, personal interests, quality of social interactions,^19^ non-tangible resources, complexity of the activities, perceived value, and regulations (internal or legal).^20^ Especially “PwD & family caregivers” seem to be faced with challenges of implementing them, what could explain why they use fewer activities. Further research is necessary to evaluate the relevance of these and other potential barriers for each activity and in general. Participants “otherwise involved” in dementia care, such as counselors and therapists, supported the use of more activities, what emphasizes their awareness of potential benefits through them.

Perceived effects were significant predictor of use. On average, our participants reported that they perceived effects for 85% of the activities, mainly on wellbeing but also on activation and improving social health. Wellbeing, or *living well with dementia*, has only recently become a central focus in dementia care. With this, a person-centered approach that goes beyond the diagnostic, therapeutic, and nursing processes has gained acknowledgement that emphasizes moments of enjoyment and meaningful activities in the lives of people with dementia. And, as our results indicate, non-medical activities make a contribution to that. Many previous studies have demonstrated positive effects of, for instance, creative arts,^21^ art-based intervention,^22^ horticultural therapy,^23^ creative dance,^24^ and music^25^ on wellbeing. Moreover, there is evidence that some leisure activities, for example, exercise,^26^ religiosity,^27^ or music^28^ can slow cognitive decline of people with dementia. Participating in activities might also counteract apathy, a symptom affecting about every fifth person with dementia.^29^ While our participants only reported to us the main effect that they perceived, regular engagement in certain activities might come with yet unknown benefits. To achieve the best possible outcome through non-medical activities, it would be valuable to selectively match the symptom profile of a person with dementia with activities that address exactly those symptoms while taking into account their personal interests and abilities. However, research on most non-medical activities is still in the beginning stages and a lot of work is needed to establish the respective effect sizes. The finding that PwD and family caregivers perceived fewer effects is puzzling. It might be due to lower expectations, less dementia-related training, or the fact that they reported only on one (their own) observation and not on observations of several patients as professional caregivers. This implicates that PwD and family caregivers may need practical information or guidance on what activities are best to engage in.

This study is not without limitations. First, even though we tried to reach as many people as possible in dementia care, those who decided to participate may not be representative for all. Unavailable information on non-responders does not allow us to conclude how response bias may have affected our results. As there were no comparable previous studies, we were not able to calculate an optimal sample size. Future studies can now do so based on our results. Second, we used open questions to assess utilization and perceived effects. We cannot exclude that participants would report also other types of effects, if they had multiple answer options to select from or had the chance to observe effects over a certain period of time. Effects perceived by stakeholder may also not correspond to changes in objectively measured assessments, which go unnoticed. Moreover, some activities naturally do not come with certain types of effects and our scores do not reflect that. As questions for assessing use and effects were conceived specifically for this study, further validation is necessary. Potential biases may further relate to our sampling strategy, placebo effects, and not controlling for confounders and findings may not be representative for other health care systems or cultural contexts. Further, using a significance level of 0.05 might come with false positives. However, we chose this level because we did not want to risk false negative findings. Further studies are necessary to validate the findings.

Our findings emphasize that many non-medical activities are being used in dementia care. The perceived increase in wellbeing for people with dementia through these activities emphasize the importance of the efforts of those involved, while more research is needed to specify the broad range of effects that can actually be achieved. Enhancing the implementation of non-medical activities to achieve benefits for a greater number of people with dementia might be challenging as, for instance in dementia care facilities, even now interventions with well-known effects (e.g., reminiscence, vocational occupation) are seldom offered.^30^ While analyses did not point out any general barrier, more research investigating person-centered barriers as well as motivating aspects is needed.

## Supplemental Material

sj-pdf-1-jpc-10.1177_21501319251390081 – Supplemental material for Non-Medical Activities in Dementia Care in Germany: Use and Experienced Effects Supplemental material, sj-pdf-1-jpc-10.1177_21501319251390081 for Non-Medical Activities in Dementia Care in Germany: Use and Experienced Effects by Francisca S. Rodriguez, Nadja Ziegert and Sabrina D. Ross in Journal of Primary Care & Community Health
