# Supplementary material for: Non-Medical Activities in Dementia Care in Germany: Use and Experienced Effects
Source: J Prim Care Community Health. 2025 Nov 4;16:21501319251390081. doi: 10.1177/21501319251390081 (PMC12586855; doi:10.1177/21501319251390081)
Supplement: sj-pdf-1-jpc-10.1177_21501319251390081 – Supplemental material for Non-Medical Activities in Dementia Care in Germany: Use and Experienced Effects [file sj-pdf-1-jpc-10.1177_21501319251390081.pdf]

Supplementary File

To the manuscript 'Non-medical activities in dementia care'

Table S1. Use of non-medical activities by group of participants (n=132).

	**People with dementia & family caregivers (n=49)**	**Caregiving professionals (n=41)**	**'Otherwise involved' (n=42)**	
	**M (SD)**	**M (SD)**	**M (SD)**	**x^2 (p)**
All non-medical activities	14.3 (4.6)	17.4 (4.7)	21.9 (6.9)	**30.945 (<0.001)**
Household activities	1.8 (1.4)	2.8 (1.5)	3.6 (1.6)	**25.818 (<0.001)**
Social activities	3.5 (1.2)	3.4 (1.9)	4.8 (1.7)	**19.689 (<0.001)**
Leisure activities	9.0 (3.2)	11.1 (3.2)	13.5 (5.1)	**24.475 (<0.001)**
	**% (n)**	**% (n)**	**% (n)**	**x^2 (p)**
**Household activities**				
Cooking	57.1% (28)	78.6% (33)	88.1% (37)	**11.929 (0.003)**
Cleaning	55.1% (27)	66.7% (28)	76.2% (32)	4.488 (0.106)
Baking	24.5% (12)	78.6% (33)	83.3% (35)	**41.359 (<0.001)**
Laundry	26.5% (13)	38.1% (16)	66.7% (28)	**15.444 (<0.001)**
Fixing broken things	18.4% (9)	19.1% (8)	42.9% (18)	**8.667 (0.013)**
**Social activities**				
Visits	95.9% (47)	80.9% (34)	95.2% (40)	**7.529 (0.023)**
Excursions	73.5% (36)	76.2% (32)	95.2% (40)	**8.032 (0.018)**
Making phone calls	71.4% (35)	78.6% (33)	92.9% (39)	**6.741 (0.034)**
Using visitation services	32.7% (16)	54.8% (23)	54.8% (23)	**6.079 (0.048)**
Receiving neighborhood assistance	40.8% (20)	26.8% (11)	64.3% (27)	**12.125 (0.002)**
Attending club meetings	20.4% (10)	19.5% (8)	52.4% (22)	**14.225 (0.001)**
Using phone services	n < 5	n < 5	19.1% (8)	**8.233 (0.016)**
Volunteering	12.2% (6)	n < 5	n < 5	**6.169 (0.046)**
**Leisure activities**				
Spending time in nature	83.7% (41)	95.2% (40)	85.2% (40)	5.042 (0.080)
Reading	79.6% (39)	92.9% (39)	90.5% (38)	4.154 (0.125)
Playing games	55.1% (27)	92.9% (39)	92.9% (39)	**26.542 (<0.001)**
Listening to music	73.5% (36)	69.1% (29)	85.7% (36)	3.452 (0.178)
Singing	51.0% (25)	78.6% (33)	95.2% (40)	**23.559 (<0.001)**
Gardening	53.1% (26)	78.6% (33)	76.2% (32)	**8.527 (0.014)**
Dancing	51.0% (25)	64.3% (27)	83.3% (35)	**10.472 (0.005)**
Going to cinema/ museum/ theater	51.0% (25)	73.8% (31)	71.4% (30)	**6.370 (0.041)**
Crafts	26.5% (13)	85.7% (36)	83.3% (35)	**44.782 (<0.001)**
Watching TV	81.6% (40)	35.7% (15)	52.4% (22)	**20.327 (<0.001)**
Puzzling	48.9% (24)	50.0% (21)	69.1% (29)	4.481 (0.106)
Attending religious events	32.7% (16)	66.7% (28)	71.4% (30)	**16.801 (<0.001)**
Drawing	14.3% (7)	66.7% (28)	80.9% (34)	**45.638 (<0.001)**
Needlework	22.5% (11)	61.9% (26)	73.8% (31)	**26.728 (<0.001)**
Physical activity	57.1% (28)	28.6% (12)	61.9% (26)	**11.088 (0.004)**
Traveling/ vacation	57.1% (28)	n < 5	40.5% (17)	**25.024 (<0.001)**
Playing musical instrument	n < 5	42.9% (18)	57.1% (24)	**25.837 (<0.001)**
Keeping pets	36.7% (18)	14.3% (6)	35.7% (15)	**6.709 (0.035)**
Foreign language	18.4% (9)	n < 5	33.3% (14)	**7.537 (0.023)**

Notes: M, mean; p, level of significance; SD, standard deviation; x^2, chi square of Kruskal-Wallis test for continuous variables and chi square test for categorical variables.

Table S2. Frequency of use of each non-medical activities and effects perceived for it (n=134).

	**Utilization**		**Effect perceived**	
	**n**	**%**	**n**	**%** §
**Household activities**				
Cooking	98	73.7	93	94.9
Cleaning	87	65.4	78	89.7
Baking	80	60.2	75	93.8
Laundry	57	42.9	49	86.0
Fixing broken things	35	26.3	34	97.1
**Social activities**				
Visits	121	90.9	111	91.7
Excursions	108	81.2	102	94.4
Making phone calls	107	80.5	90	84.1
Using visitation services	62	46.6	56	90.3
Receiving neighborhood assistance	58	43.9	55	94.8
Attending club meetings	40	30.3	37	92.5
Using phone services	12	9.0	11	91.7
Volunteering	8	6.0	0	0.0
**Leisure activities**				
Spending time in nature	121	90.9	111	91.7
Reading	116	87.2	96	82.8
Playing games	105	78.9	93	88.6
Listening to music	101	75.9	81	80.2
Singing	98	73.7	91	92.9
Gardening	91	68.4	74	81.3
Dancing	87	65.4	79	90.8
Going to cinema/ museum/ theater	86	64.7	80	93.0
Crafts	84	63.2	74	88.1
Watching TV	77	57.9	58	75.3
Puzzling	74	55.6	54	73.0
Attending religious events	74	55.6	58	78.4
Drawing	69	51.9	58	84.1
Needlework	68	51.1	54	79.4
Physical activity	66	49.6	60	90.9
Traveling/ vacation	48	36.1	40	83.3
Playing musical instrument	46	34.6	41	89.1
Keeping pets	39	29.3	35	89.7
Foreign language	27	20.3	22	81.5

Notes: §, percentage of those who reported engaging in the activity; m, means; n, number of participants; SD, standard deviation.

Table S3. Percentage of activities for which an effect was perceived (n=132).

	**People with dementia & family caregivers (n=49)**	**Caregiving professionals (n=41)**	**'Otherwise involved' (n=42)**	
	**M (SD)**	**M (SD)**	**M (SD)**	**x² (p)**
**All non-medical activities**				
Any effect	76.7% (31.2%)	86.7% (25.2%)	93.3% (24.7%)	**15.265 (<0.001)**
Wellbeing	44.9% (27.3%)	51.9% (23.1%)	52.9% (29.6%)	2.464 (0.292)
Appreciation	2.1% (6.5%)	4.5% (8.4%)	3.4% (7.2%)	2.305 (0.316)
Improved social health	17.2% (14.2%)	28.9% (20.5%)	26.2% (20.2%)	**8.674 (0.013)**
Preserving abilities	2.7% (6.9%)	5.8% (7.9%)	7.9% (8.9%)	**8.751 (0.013)**
Improved cognition	0.5% (1.9%)	0.9% (1.9%)	1.3% (2.2%)	2.317 (0.314)
Relaxation	2.8% (4.1%)	4.7% (5.9%)	2.7% (4.9%)	2.682 (0.262)
Activation	17.1% (18.9%)	29.3% (22.7%)	26.9% (31.1%)	**7.002 (0.030)**
**Leisure activities**				
Any effect	76.7% (36.4%)	86.5% (28.1%)	92.1% (31.2%)	**15.372 (<0.001)**
Wellbeing	45.7% (29.9%)	52.7% (24.1%)	58,9% (34.6%)	5.638 (0.059)
Appreciation	0.3% (1.8%)	1.3% (3.3%)	0.6% (2.2%)	0.990 (0.609)
Improved social health	7.3% (8.8%)	18.1% (20.4%)	11.9% (20.2%)	**7.075 (0.029)**
Preserving abilities	0.7% (2.5%)	1.4% (3.8%)	0.8% ('2.7%)	0.130 (0.937)
Improved cognition	0.0% (0.0%)	0.0% (0.0%)	0.0% (0.0%)	-
Relaxation	4.4% (7.0%)	6.9% (8.9%)	4.3% (7.4%)	2.176 (0.337)
Activation	18.9% (21.6%)	30.1% (23.1%)	34.5% (38.2%)	5.393 (0.067)
**Household activities**				
Any effect	59.5% (47.1%)	80.8% (40.2%)	85.7% (35.4%)	**6.628 (0.036)**
Wellbeing	29.9% (40.9%)	45.3% (49.2%)	51.3% (46.8%)	3.637 (0.162)
Appreciation	4.6% (20.2%)	10.5% (30.0%)	8.6% (23.9%)	0.454 (0.797)
Improved social health	11.9% (28.9%)	23.1% (40.8%)	11.7% (29.6%)	1.116 (0.558)
Preserving abilities	11.2% (31.1%)	26.4% (41.4%)	39.8% (47.6%)	**7.626 (0.022)**
Improved cognition	0.0% (0.0%)	0.0% (0.0%)	0.0% (0.0%)	-
Relaxation	0.0% (0.0%)	0.0% (0.0%)	0.0% (0.0%)	-
Activation	2.0% (14.3%)	24.9% (39.5%)	18.6% (37.3%)	5.744 (0.057)
**Social activities**				
Any effect	82.0% (32.0%)	73.1% (40.6%)	90.5% (26.3%)	4.059 (0.131)
Wellbeing	43.9% (39.8%)	31.2% (41.6%)	29.9% (37.2%)	4.111 (0.128)
Appreciation	2.7% (10.9%)	7.2% (19.6%)	6.6% (19.2%)	0.511 (0.775)
Improved social health	40.3% (37.2%)	54.9% (45.4%)	66.8% (38.7%)	**10.832 (0.004)**
Preserving abilities	0.0% (0.0%)	0.0% (0.0%)	0.0% (0.0%)	-
Improved cognition	0.0% (0.0%)	0.0% (0.0%)	0.0% (0.0%)	-
Relaxation	0.0% (0.0%)	0.0% (0.0%)	0.0% (0.0%)	-
Activation	15.9% (26.9%)	18.5% (33.4%)	21.0% (38.9%)	0.021 (0.989)

Notes: M, mean; p, level of significance; SD, standard deviation; x^2, chi square of Kruskal-Wallis test.
